# Human Wharton's Jelly Stem Cell (hWJSC) Extracts Inhibit Ovarian Cancer Cell Lines OVCAR3 and SKOV3 *in vitro* by Inducing Cell Cycle Arrest and Apoptosis

**DOI:** 10.3389/fonc.2018.00592

**Published:** 2018-12-07

**Authors:** Gauthaman Kalamegam, Khalid Hussein Wali Sait, Farid Ahmed, Roaa Kadam, Peter Natesan Pushparaj, Nisreen Anfinan, Mahmood Rasool, Mohammad Sarwar Jamal, Muhammed Abu-Elmagd, Mohammed Al-Qahtani

**Affiliations:** ^1^Center of Excellence in Genomic Medicine Research, King Abdulaziz University, Jeddah, Saudi Arabia; ^2^Faculty of Medicine, Asian Institute of Medicine, Science and Technology (AIMST) University, Bedong, Malaysia; ^3^Department of Obstetrics and Gynaecology, Faculty of Medicine, King Abdulaziz University, Jeddah, Saudi Arabia; ^4^King Fahad Medical Research Centre (KFMRC), King Abdulaziz University, Jeddah, Saudi Arabia

**Keywords:** stem cells, cancer stem cells, cell cycle, cell migration, tumor spheres, gene expression, OVCAR3, SKOV3

## Abstract

Ovarian cancer is a highly lethal and the second highest in mortality among gynecological cancers. Stem cells either naïve or engineered are reported to inhibit various human cancers in both *in-vitro* and *in-vivo*. Herein we report the cancer inhibitory properties of human Wharton's jelly stem cell (hWJSC) extracts, namely its conditioned medium (hWJSC-CM) and cell lysate (hWJSC-CL) against two ovarian cancer cell lines (OVCAR3 and SKOV3) *in-vitro*. Cell metabolic activity assay of OVCAR3 and SKOV3 cells treated with hWJSC-CM (12.5, 25, 50, 75, 100%) and hWJSC-CL (5, 10, 15, 30, and 50 μg/ml) demonstrated concentration dependent inhibition at 24–72 h. Morphological analysis of OVCAR3 and SKOV3 cells treated with hWJSC-CM (50, 75, 100%) and hWJSC-CL (15, 30, and 50 μg/ml) for 24–72 h showed cell shrinkage, membrane damage/blebbings and cell death. Cell cycle assay demonstrated an increase in the sub-G1 and G2M phases of cell cycle following treatment with hWJSC-CM (50, 75, 100%) and hWJSC-CL (10, 15, and 30 μg/ml) at 48 h. Both OVCAR3 and SKOV3 cells demonstrated mild positive expression of activated caspase 3 following treatment with hWJSC-CM (50%) and hWJSC-CL (15 μg/ml) for 24 h. Cell migration of OVCAR3 and SKOV3 cells were inhibited following treatment with hWJSC-CM (50%) and hWJSC-CL (15 μg/ml) for 48 h. Tumor spheres (TS) of OVCAR3 and SKOV3 treated with hWJSC-CM (50, 75, 100%) and hWJSC-CL (10, 15, 30 μg/ml) for 48 h showed altered surface changes including vacuolations and reduction in size of TS. TS of OVCAR3 and SKOV3 also showed the presence of few ovarian cancer stem cells (CSCs) in minimal numbers following treatment with hWJSC-CM (50%) or hWJSC-CL (15 μg/ml) for 48 h. Real-time gene expression analysis of OVCAR3 and SKOV3 treated with hWJSC-CM (50%) or hWJSC-CL (15 μg/ml) for 48 h demonstrated decreased expression of cell cycle regulatory genes (cyclin A2, Cyclin E1), prostaglandin receptor signaling genes (EP2, EP4) and the pro-inflmmatory genes (IL-6, TNF-α) compared to untreated controls. The results indicate that hWJSC-CM and hWJSC-CL inhibit ovarian cancer cells at mild to moderate levels by inducing cellular changes, cell cycle arrest, apoptosis, decreasing the expression of CSC markers and related genes regulation. Therefore, the stem cell factors in hWJSCs extracts can be useful in cancer management.

## Introduction

Ovarian cancer is the most lethal and the second highest in mortality compared to other gynecological cancers. It is estimated the nearly 22,000 ovarian cancer cases are diagnosed each year and the annual death rates are over 14,000 deaths (1). Given the slow progression of the disease, ovarian cancer is often diagnosed at later stages when metastases have already occurred. Furthermore, subclinical metastasis associated with ovarian carcinoma renders their localization within the peritoneal cavity, and the disease escapes early detection. Lack of suitable biological markers except for the commonly screened CA125 also may be one of the factors for late diagnosis. CA125 is known to be expressed by 85% of serous ovarian tumors, 70% of the undifferentiated tumors and only rarely by mucinous tumors (2).

Debulking or cytoreductive surgery continue to be the main stay of treatment prior to institution of adjuvant chemotherapy with platinum/taxane based drugs. The prognosis of ovarian cancer is however dependent upon its stage at detection and its histological type. Early detection and well-differentiated stage-I epithelial ovarian carcinoma (EOC) have a 5-year survival of 90%. Chemotherapy with platinum/taxane based drugs are effective in at least 70% of the cases, if detected early. Majority of the ovarian cancer patients respond initially well to chemotherapy comprising of 6–9 cycles of carboplatin and taxane based drugs (3). However, the major drawback with ovarian cancer prognosis is the emergence of multi-drug resistance (MDR), which then lead to relapse of the disease within a short span of 1–2 years (4).

One emerging theory with drug resistant ovarian cancer is the presence of cancer initiating cells (CICs) or the cancer stem cells (CSCs) which is responsible for relapse of the malignant tumor phenotype despite earlier cytoreductive surgery. These CICs/CSCs are thought to have acquired features that confer chemoresistance, following expression of membrane efflux reporters, low mitotic index and enhanced deoxyribonucleic acid (DNA) repair mechanisms and many different methods to identify and isolate these so called CICs are reported (5). The identification of both SP cells and ALDH bright cells were found to be associated in tumor relapses as only few cells were necessary for tumor initiation; had higher tumor sphere forming ability and demonstrated high expression of the pluripotent gene namely, sex determining region Y-box 2 (SOX2) (6). Given the fact that complete remission of ovarian cancers with the existing conventional therapies is still lacking, active research in the following areas such as, targeting the MDR with more specific small molecular inhibitors; targeting of the CSCs and deregulated non-coding RNA (ncRNA); modulating the signaling pathways associated with apoptosis/autophagy are being vigorously researched (7).

Interestingly, earlier studies have reported that mesenchymal stem cells (MSCs) have anticancer effects. MSCs *per se* or their secretome have been reported to impart anticancer effects (8). Human Wharton's Jelly stem cells (hWJSCs) derived from within the Wharton's jelly of the umbilical cord (which is usually discarded at birth) is fetal in origin, and therefore have the properties of both embryonic and mesenchymal stem cells (9). Various research groups have identified that the tumor inhibition properties of hWJSCs spans across many different human cancers (8, 10–12). Furthermore, unlike MSCs derived from other sources, the hWJSCs do not cause tumor *in vivo* in immunodeficient mice (13).

Given the beneficial properties of hWJSCs, we evaluated the anticancer properties of hWJSCs on two commercial ovarian carcinoma cell lines (OVCAR3 and SKOV3) *in vitro* using the following parameters namely, cell morphology, cell metabolic activity, cell cycle, cell death, caspase 3 assay, cell migration, CSCs inhibition, tumor sphere (TS) inhibition and gene expression related to cell cycle, prostaglandin receptor signaling and inflammation.

## Materials and Methods

### Ethical Approval

The ethical approval for derivation and use of derived human Wharton's Jelly stem cells (hWJSCs), and the commercial human ovarian cancer cell lines (OVCAR3 and SKOV3) was obtained from the Bioethics Committee of the King Abdulaziz University *vide* approval number [33-15/KAU], with written informed consent from all subjects. All subjects gave written informed consent in accordance with the Declaration of Helsinki.

### Establishment of Human Wharton's Jelly Stem Cells (hWJSCs)

Human umbilical cords (*n* = 10) were obtained following informed consent from patients undergoing full-term derlivery at the Department of Obstetrics and Gynecology, King Abdulaziz University Hospital (KAUH). The umbilical cord was transferred in a sterile container containing Hanks balanced salt solution (HBSS) and antibiotics and processed within 6 h. Derivation of hWJSCs were done according to the protocol published earlier (14, 15). Briefly, the umbilical cord was cut into pieces of ~2 cm and opened length wise. The blood vessels were removed and the opened side exposed to an ezyme cocktail containing collagenase type-I (2 mg/mL), collagenase type-IV (2 mg/mL) and hyaluronidase (100 IU) for 30 min. The enzyme ativity was blocked by addition of medium containing 10% fetal bovine serum (FBS), and the matrix contents were gently scraped and the medium containing cells and matrix substance was centrifuged at 500 g × 5 min. The cell pellet was washed twice with phosphate bufered saline (PBS^−^) devoid of calcium chloride and magensium and centrifuged again. The resultant pellet was resuspended in culture media comprised of DMEM high glucose (DMEM-HG), supplemented with 10% FBS, 2 mM Glutamax, 1% non-essential aminoacids (NEAA), basic fibroblast growth factor (bFGF) 16 ng/mL and 1% antibiotics [pencillin (50 IU/ml), streptomycin (50 μg/ml)] and incuabted at standard culture conditions of 37°C in a 5% CO_2_ incubator. The cultures were left undisturbed until cell growth was evident, except for gentle changes of growth media every 72 h. The deirved cells were tested for their biological and stemness properties before being utilized in the study.

### CD Marker Analysis

The derived hWJSCs were initially analyzed for the presence of MSCs related surface CD markers using fluorescent activated cell sorting (FACS) as reported earlier (14). Briefly, hWJSCs were trypsinized and centrifuged (1000 rpm × 5 min) and the cell pellet was gently resuspended in 5 ml of PBS^−^ to obtain single cell suspension. The cells were counted, aliquoted (1 × 10^5^ cells/15 ml tube per treatment condition) and blocked with 100 μl of 3% FBS to prevent non-specific binding. CD marker cocktails (containing 5 μl per individual CD marker) were freshly prepared and used to assess the MSCs related CD markers (Miltenyi Biotec) as follows: MSC isotype cocktail (negative control); MSC positive cocktail 1 (containing CD45-APC, CD105-FITC, CD73-PERCP) and MSC positive cocktail 2 (containing CD29-PERCP, CD34-PE, CD44-PE-CY, CD90-FITC). Respective CD markers cocktail were added to the different samples and incubated in the dark at 4°C for 20–30min. The cells were washed twice with 3% FBS buffer and centrifuged (1000 rpm × 5 min). The resultant pellet was resuspended in 500 μl of 3% FBS and analyzed using FACS (FACS Aria II, BD Biosciences).

### Preparation of hWJSC-Conditioned Media (hWJSC-CM)

Early passages of hWJSCs were grown under standard culture conditions and the media changed every 48 h. When the cells were 70% confluent, the spent culture media was replaced with fresh media and the cells cultured for upto 72 h. The hWJSC-conditioned media (hWJSC-CM) was then harvested, filter sterilized using 0.2 μm syringe filters and aliquots stored at 4°C until use in experiments (16). Neat samples of hWJSC-CM represented 100%, while the other percentages of hWJSC-CM was prepared by diluting the neat hWJSC-CM with basal culture medium.

### Preparation of hWJSC-Cell Lysate (hWJSC-CL)

Derived hWJSCs were grown under standard culture conditions and the media changed every 48 h. At confluence the cells were trypsinized and centrifuged (1000 rpm × 5 min) and the pellet washed twice in PBS^−^ and centrifuged again. The resultant cell pellet was lysed in cell lysis buffer (Sigma, St Louis, MO). Protease inhibitor cocktail (Sigma, ST Louis, MO) was added to the cell lysis buffer prior to use. The cells were pipetted up and down to lyse the membranes and release the cellular contents. The cells in suspension were incuabted in a rocker platform for 15 min followed by centrifugation (15,000 rpm × 15 min). The clear supernatant was collected, aliquoted and stored at 4°C until use in experiments. The total protein content was quantified using Nanodrop (Nanodrop technologies, Wilmington, CA) (16).

### Cutlure of OVCAR3 and SKOV3

Human commercial ovarian cancer cell lines (OVCAR3 and SKOV3) were purchased from the European Collection of Authenticated Cell Cultures (ECACC). OVCAR3 cells were cultured in Low Glucose Dulbecco's Modified Eagles Medium (DMEM-LG) and SKOV3 cells were cultured in Roswell Park Memorial Institute (RPMI) 1640. Both media were supplemented with 10% FBS, 2 mM Glutamax and 1% penicillin-streptomycin. Both OVCAR3 and SKOV3 cells were rapidly thawed in 37°C waterbath using standard cell thawing procedure and cultured at optimal conditions of 37°C in a 5% CO_2_ incubator. Both OVCAR3 and SKOV3 cells represent serous adenocarcinoma having low and high invasion potential, respectively. Both cell types exhibit drug resistance, presence of hormone receptors and tumorigenic potential, and are therefore commonly used in *in vitro/in vivo* research studies.

### Cell Metabolic Activity

The hWJSCs and commercial ovarian cancer cell lines (OVCAR3, SKOV3) were plated at a seeding density of 2 × 10^4^ cells/well of a 24 well plate and allowed to attach overnight. The culture media was then replaced with media containing different concentrations of either hWJSC-CM (12.5, 25, 50, 75, 100%); hWJSC-CL (5, 10, 15, 30, 50 μg/ml); paclitaxel (2.5, 5, 10, 20, 30 nM) or doxorubicin (3, 10, 30, 100, and 300 nM) for 24, 48 and 72 h. Control cells were cultured in normal culture medium without any pharmacological agents. The cell metabolic activity/inhibition following different experimental conditions were analyzed using MTT assay according to manufacturer's instructions. Briefly, the culture media was removed and 200 μl of fresh media containing 10 μl of MTT reagent (3-(4, 5-dimethylthiazolyl-2)-2, 5-diphenyltetrazolium bromide; Sigma, MO) was added and incubated under standard culture conditions for upto 4 h. The media was removed and 200 μl of solubilization reagent was added. Absorbance was obtained at 570 nM with a reference wavelength of 630 nM using SpectraMax i3 Multimode Reader (Molecular Devices, USA).

### Cell Morphology

OVCAR3 and SKOV3 cells were plated at a seeding density of 2 × 10^4^ cells/well of a 24 well plate and tested with hWJSC-CM (50, 75, 100%); hWJSC-CL (10, 15, 30 μg/ml); paclitaxel (2.5, 5, 10, 20, 30 nM) or doxorubicin (3, 10, 30, 100, 300 nM). Both controls and experimental groups were then cultured at 37°C in a 5% CO_2_ incubator for up to 24–72 h. Cell morphology including any visible changes following experimental procedures were imaged every 24 h using inverted phase contrast microscopy (Nikon Instruments, Japan).

### Cell Cycle Assay

OVCAR3 and SKOV3 cells were plated at a seeding density of 1 × 10^5^ cells/T25 cm^2^ tissue culture flask and tested with hWJSC-CM (50, 75, 100%); hWJSC-CL (10, 15, 30 μg/ml); paclitaxel (2.5, 5, 10 nM) or doxorubicin (10, 30, 100 nM). Briefly, the control and treated cells were fixed with ice-cold 70% ethanol for 2–3 h by dropwise addition to avoid clumping of cells. The fixed cells were washed with PBS^−^ and stained with 50 μg/ml propidium iodide (PI) in PBS^−^ containing 0.1% TritonX-100 and 50 μg/ml RNAse-A. The cells were analyzed using FACS Aria III flow cytometer (BD Biosciences, San Jose, CA) and results computed using FACSDiva^TM^ software (BD Biosciences, San Jose, CA).

### Caspase Activation Assay

Detection of cleaved caspase 3 was done using fluorescin isothiocyante (FITC) conjugated active caspase 3 antibody according to the manufacturer's instructions (BD Biosciences, San Jose, CA). Briefly, both OVCAR3 and SKOV3 cells were plated at a seeding density of 1 × 10^5^ cells/T25 cm^2^ tissue culture flask and allowed to attach overnight. Both OVCAR3 and SKOV3 cells were then treated with hWJSC-CM (50%), hWJSC-CL (15 μg/ml), paclitaxel (5 nM) or doxorubicin (30 nM) for 24 h under standard culture conditions. Following the treatment period the cells were trypsinized, centrifuged (1000 rpm × 5 min) and the pellet washed with ice-cold PBS. The cells were again centrifuged and the pellet was resuspended in 0.5 ml of Cytofix/Cytoperm solution and incubated on ice for 20 min. The cells were then washed twice with Perm/Wash buffer (1x) and incubated with 5 μl of FITC conjugated rabbit anti-caspase 3 antibody for 30 min at room temperature. The cells were washed again and resuspended in 300 μl buffer and analyzed using FACS Aria III flow cytometer (BD Biosciences, San Jose, CA) and results computed using FACSDiva^TM^ software (BD Biosciences, San Jose, CA).

### Cell Migration Assay

OVCAR3 and SKOV3 were initially cultured in respective serum-free culture medium for 24 h. The serum-starved cells were then seeded at a density of 1 × 10^5^ cells/well and cultured in the upper chamber of 24-well transwell (8 mm pore size) culture plates (Corning Costar Corporation, Cambridge, MA) in serum-free medium. The lower chamber was filled with respective culture medium containing either hWJSC-CM (50%) or hWJSC-CL (15 μg/ml) and the culture plate was incubated under standard culture conditions of 37°C in a 5% CO_2_ incubator. Cell migration from upper to lower chambers was evaluated after 24 h. The culture inserts were treated with the cell dissociation buffer in new wells of 24-well plates, and further washed with respective media to ensure that all the cells attached to the lower surface of the transwell membrane were completely dislodged into the new well. The migrated cells were quantified following treatment with MTT reagent and measuring their absorbance at 570 nM with a reference wavelength of 630 nM using SpectraMax i3 Multimode Reader (Molecular Devices, USA).

### Tumor Sphere (TS) Assay

OVCAR3 and SKOV3 cells were seeded at a seeding density of 1.5 × 10^5^ cells/well in 6-well ultralow attachment plates (Corning Inc) in serum-free DMEM/F12 medium with 1% of methylcellulose supplemented with 1% penicillin-streptomycin, 20 nM progesterone, 100 μM putrescine, 1% insulin-transferrin-selenium, 10 ng/ml b-FGF and 10 ng/ml human rEGF. After 7 to 12 days of culture, the generated TS were imaged using inverted phase contrast microscope (Nikon Instruments, Japan). Tumor spheres were then filtered to separate uniform sized TS of more than 100 μm size and equal numbers plated in a new 6-well ultralow attachment plates. These TS were then exposed to experimental agents hWJSC-CM (50, 75, 100%); hWJSC-CL (10, 15, 30 μg/ml); paclitaxel (2.5, 5, 10 nM) or doxorubicin (10, 30, 100 nM) and any changes in morphology were imaged using inverted phase contrast microscopy (Nikon Instruments, Japan).

### Cancer Stem Cell (CSC) Markers Screening

The TS generated from OVCAR3 and SKOV3 as above were plated in 24 well tissue culture plates to have approximately equal sizes and numbers of spheroids per well. The TS were then treated with hWJSC-CM (50%), hWJSC-CL (15 μg/ml) or paclitaxel (10 nM) and cultured for 48 h under standard culture conditions of 37°C in a 5% CO_2_ incubator. The untreated TS of both OVCAR3 and SKOV3 served as controls. Following the study period both experimental and control TS were treated with trypsin to enable cell dissociation and was neutralized with media containing 10% FBS. The dissociated cells from each experimental condition were gently mixed to obtain single cell suspension and centrifuged (1000 rpm × 5 min), washed twice with PBS^−^ and centrifuged again. The resultant cell pellet was then resuspended in PBS^−^, cells counted and 1 × 10^5^ cells per tube were used for staining with CSCs related CD marker cocktails (pooled based on fluorescent markers with different wavelengths). Unstained cells were used for isotype controls. Following respective staining for 30 min on ice, the cells were washed with PBS^−^ containing 3% FBS to remove unbound antibodies/labels. The final cell pellet from both control and experimental samples were reconstituted in 100 μl of buffer and analyzed using FACS Aria III flow cytometer (BD Biosciences, San Jose, CA). The results were then computed using FACSDiva^TM^ software (BD Biosciences, San Jose, CA).

### Gene Expression Analysis

Both OVCAR3 and SKOV3 cells treated with hWJSC-CM (50%), hWJSC-CL (15 μg/ml), paclitaxel (10 nM) and doxorubicin (30 nM) were analyzed for cell cycle, pro-inflammatory and prostaglandin receptor related gene expression using quantitative real-time polymerase chain reaction (qRT-PCR). Briefly, the total ribonucleic acid (RNA) was extracted from the control and treated OVCAR3 and SKOV3 cells using Pure Link® RNA Mini Kit (Ambion^TM^, Thermo Fischer Scientific) according to the manufacturer's protocol. First-strand cDNA synthesis was carried out using random hexamers (High Capacity cDNA Reverse Transcription Kit, Applied Biosystems) and on column deoxyribonuclease (DNase-I) treatment was included in the protocol. Genes related to cell cycle (Cyclin A2, Cyclin E1), prostaglandin receptor signaling (EP2, EP4) and inflammation (TNF-α, IL-6) that are involved in cell cycle check, angiogenesis, cancer growth and progression were studied. Primers were taken from earlier published reports (17, 18, 19) and the primer sequences are given in Table 1. The qRT-PCR analysis was performed using the ABI StepOnePlus^TM^ Real-Time PCR System (Applied Biosystems) using SYBR green dye master mix and relative quantification was performed using the comparative 2 –ΔΔCt method which analyzes the relative change in gene expression based on Ct values (fluorescence denoting cDNA copy numbers) of housekeeping gene and gene of interest.

**Table 1:** The genes and primer sequences used for quantitative real time PCR.

**Gene**	**Primer sequence**
GAPDH	F: 5′-ACCACAGTCCATGCCATCAC-3′
	R: 5′-TCCACCACCCTGTTGCTGTA-3′
Cyclin A2	F: 5′-CCTCTCCTCCATGTCTGT GTI-AAG-3′
	R: 5′-GTGCTCCATICTCAGAACCTG CTI-3′
Cyclin El	F: 5′-TGCAGATCGCAGAGCTICTA-3′
	R: 5′-CTTICTTTGCTIGGGCTITG-3′
EP2	F: 5′-GCCACGATGCTCATCCTCTICGCC-3′
	R: 5′-CTIGTGTTCTIAATGAAATCCGAC-3′
EP4	F: 5′-TGGTATGTGGGCTGGCTG-3′
	R: 5′-GAGGACGGTGGCGAGAAT-3′
TNF-α	F: 5′-GGT-GCTIGT-TCC-TCA-GCC-TC-3′
	R: 5′-CAG-GCA-GAAGAG-CGT-GGT-G-3′
IL-6	F: 5′-CCACTCACCTCTICAGAA-3′
	R: 5′-GCGCAAAATGAGATGAGT-3′

*F, Forward primer; R, Reverse primer*.

### Statistical Analysis

The differences observed between treated and control cell numbers, cell migration and gene expression assays were analyzed using the Students *t*-test with the statistical package for Social Sciences (SPSS 13). The results were expressed as mean ± standard error of the mean (SEM) from three different replicates for individual assays and a value of *p* < 0.05 was considered to be statistically significant.

## Results

### Derivation and Culture of hWJSCs

**Figure 1:**
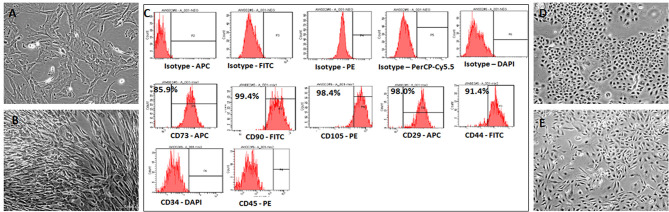
Representative phase contrast images of the human umbilical cord Wharton's Jelly Stem Cells (hWJSCs) in early and late cultures **(A,B)**. The monolayer of hWJSCs shows the characteristic appearance as short fibroblasts in early passages, which become long fibroblasts in later passages. **(C)** Representative histogram of the MSC related CD surface markers on hWJSCs using Flrorescent activated cell sorting (FACS). Top panel **(A)** shows the respective isotype controls. The middle panel **(B)** shows the positive MSC related surface CD markers namely CD73, CD90, CD105, CD29, and CD44. The bottom panel **(C)** shows the MSC negative surface CD markers namely CD34 and CD45. **(D,E)** Representative phase contrast images of the human ovarian cancer cell lines OVCAR3 **(D)** and SKOV3 **(E)** in culture showing the characteristic epitheloid cells.

The hWJSCs were derived from 10 human umbilical cords and the primary cultures of hWJSCs were characterized for their stemness and biological properties. The hWJSCs appeared as short fibroblasts in initial passages which transformed to long fbroblastic shape with subsesequent passages (Figures 1A,B). The hWJSCs expressed MSCs related CD markers namely CD29, CD44, CD73, CD90, and CD105 and their expression levels were more than 90% in almost all the derived hWJSCs. These cells were also negative for the haematopoietic stem cell markers namely CD34 and CD45 (Figure 1C).

### Culture of Commercial Ovarian Cancer Cell Lines OVCAR3 and SKOV3 Cells

The human ovarain cancer cell lines OVCAR3 and SKOV3 were immediately thawed using standard protocol and cultured using respective media. Both cell lines demonstated their characteristic epithelial morphology in culture (Figures 1D,E).

### MTT Assay of OVCAR3 Cells Treated With hWJSC-CM and hWJSC-CL

**Figure 2:**
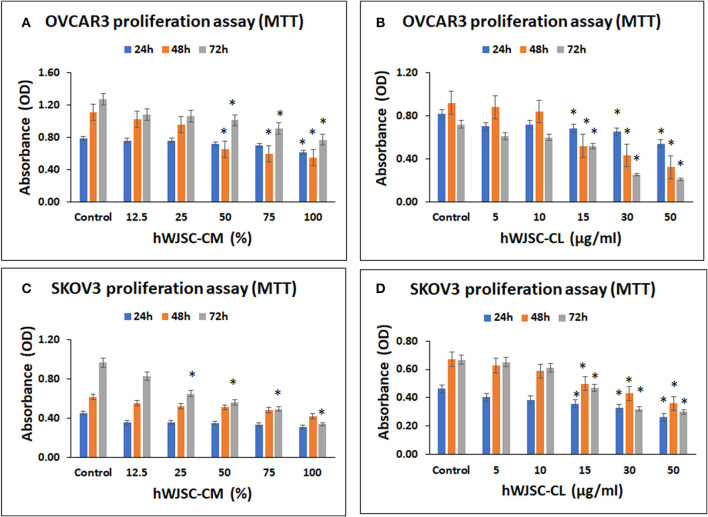
Cell metabolic activity assay of OVCAR3 cells treated with hWJSC-CM **(A)**; OVCAR3 cells treated with hWJSC-CL **(B)**; SKOV3 cells treated with hWJSC-CM **(C)**; and SKOV3 cells treated with hWJSC-CL **(D)**. The hWJSC-CM and hWJSC-CL inhibited both OVCAR3 and SKOV3 cells more at higher concentrations and duration. The values are expressed as mean ± SEM of three different experiments. *indicates statistical significance (*p* < 0.05) compared to the control. hWJSC-CM, human Wharton's jelly stem cells conditioned medium; hWJSC-CL, human Wharton's jelly stem cell lysate.

OVCAR3 cells demonstrated concentration dependent decrease in cell metabolic activity following treatment with hWJSC-CM and hWJSC-CL at 24–72 h. The mean decreases in OVCAR3 cell metabolic activity following treatment with hWJSC-CM at different concentrations (12.5, 25, 50, 75, 100%) were 3.66, 3.66, 8.44, 10.99, and 22.43% at 24 h; 7.78, 13.20, 41.43, 46.65, and 50.59% at 48 h, and 14.96, 16.54, 20.47, 28.35, and 55.12% at 72 h (Figure 2A). The mean decreases obtained in OVCAR3 cells following treatment with hWJSC-CL at different concentrations (5, 10, 15, 30, and 50 μg/ml) were 14.63, 12.20, 17.07, 20.73, and 34.15% at 24 h; 4.35, 8.70, 43.48, 53.26, and 65.22% at 48 h, and 15.04, 16.62, 27.86, 65.18, and 70.75% at 72 h (Figure 2B). The mean decreases observed in OVCAR3 cells with hWJSC-CM (100%) at 24 h; hWJSC-CM (50, 75, 100%) at 48 and 72 h; and hWJSC-CL (15, 30, 50 μg/ml) at 24, 48, and 72 h were statistically significant (*P* < 0.05) compared to the control.

### MTT Assay of SKOV3 Cells Treated With hWJSC-CM and hWJSC-CL

SKOV3 cells demonstrated concentration dependent decrease in cell metabolic activity following treatment with hWJSC-CM and hWJSC-CL at 24–72 h. The mean decreases obtained in SKOV3 cells following treatment with hWJSC-CM at different concentrations (12.5, 25, 50, 75, 100%) were 20.52, 20.52, 22.73, 25.86, 31.61% at 24 h; 10.34, 15.62, 17.07, 21.27, 31.67% at 48 h and 14.20, 32.81, 42.06, 49.03, 65.00% at 72 h (Figure 2C). The mean decreases in SKOV3 cells following treatment with hWJSC-CL at different concentrations (5, 10, 15, 30, 50 μg/ml) were 12.64, 16.82, 22.69, 29.03, 43.36% at 24 h; 6.45, 12.21, 25.48, 35.92, 46.35% at 48 h and 2.28, 8.25, 29.54, 52.02, 55.02% at 72 h (Figure 2D). The mean decreases in SKOV3 cells with higher concentrations of hWJSC-CM (25, 50, 75,100%) at 72 h and hWJSC-CL (15, 30, 50 μg/ml) at 24, 48, and 72 h were statistically significant (*p* < 0.05) compared to the control.

### MTT Assay of OVCAR3 and SKOV3 Cells Treated With Paclitaxel

**Figure 3:**
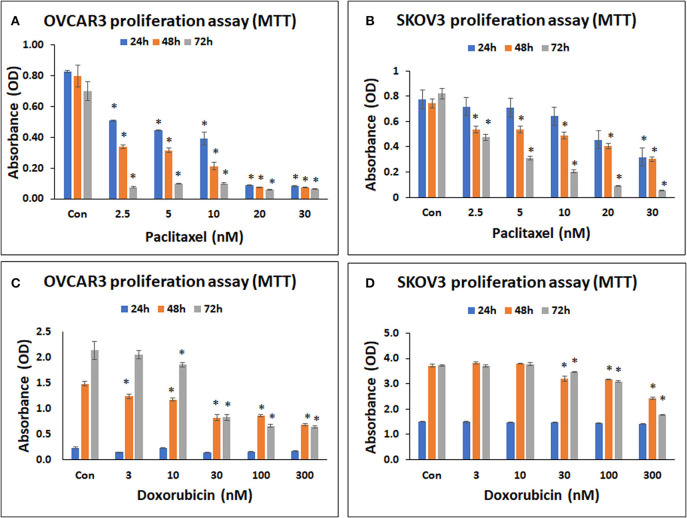
Cell metabolic activity assay of OVCAR3 cells treated with paclitaxel **(A)**; SKOV3 cells treated with paclitaxel **(B)**; OVCAR3 cells treated with doxorubicin **(C)** and SKOV3 cells treated with doxorubicin **(D)**. The standard anticancer agents (paclitaxel and doxorubicin) demonstrated significant inhibition in both OVCAR3 and SKOV3 cell metabolic activity with increasing drug concentrations and time. The values are expressed as mean ± SEM of three different experiments. *indicates statistical significance (*p* < 0.05) compared to the control.

Both OVCAR3 and SKOV3 cells demonstrated concentration dependent decrease in cell metabolic activity following treatment with paclitaxel at 24–72 h. The mean decreases obtained in OVCAR3 cells following treatment withpaclitaxel at different concentrations (2.5, 5, 10, 20, 30 nM) were 35.86, 46.22, 52.46, 89.24, 90.12% at 24 h; 57.51, 60.53, 73.29, 90.40, 90.64% at 48 h and 89.10, 85.98, 85.76, 91.21, 90.56% at 72 h (Figure 3A). The mean decreases obtained in SKOV3 cells following treatment with paclitaxel were 7.48, 8.51, 17.15, 41.33, 58.93% at 24 h; 27.34, 28.01, 34.14, 45.45, 59.33% at 48 h and 41.95, 62.26, 74.82, 88.85, 93.29% at 72 h (Figure 3B). The mean decreases in OVCAR3 cells for all concentrations of paclitaxel (2.5, 5, 10, 20, 30 nM) at 24, 48, and 72h and the mean decreases in SKOV3 cells with paclitaxel (30 nM at 24 h; 2.5, 5, 10, 20, 30 nM at 48 and 72 h) were statistically significant (*p* < 0.05) compared to the control.

### MTT Assay of OVCAR3 and SKOV3 Cells Treated With Doxorubicin

Both OVCAR3 and SKOV3 cells demonstrated concentration dependent decrease in cell metabolic activity following treatment with doxorubicin at 24–72 h. The mean decreases obtained in OVCAR3 with doxorubicin at different concentrations (3, 10, 30, 100, 300 nM) were 35.64, 3.41, 41.64, 32.27, 26.92% at 24 h; 16.53, 20.90, 44.75, 41.84, 53.98% at 48 h, and 4.11, 13.18, 61.30, 69.09, 69.92% at 72 h (Figure 3C). The mean decreases obtained in SKOV3 cells were 0.64, 0.85, 1.52, 3.71, 6.34% (for 3, 10, 30, 100, 300 nM, respectively) at 24 h; 13.82, 14.84, 34.74% (for 30, 100, 300 nM, respectively) at 48 h, and 0.63, 6.99, 17.22, 52.67% (for 3, 30, 100, 300 nM, respectively) at 72 h (Figure 3D). The mean decreases in OVCAR3 cells with doxorubicin concentrations 3, 10, 30, 100, 300 nM at 48 h as well as the mean decreases with 10, 30, 100, 300 nM at 72 h were statistically significant (*P* < 0.05) compared to the control. The mean decreases in SKOV3 with doxorubicin concentrations 30, 100, 300 nM at 48 and 72 h were statistically significant (*P* < 0.05) compared to the control.

### Cell Morphology of OVCAR3 and SKOV3 Cells With hWJSC-CM and hWJSC-CL

**Figure 4:**
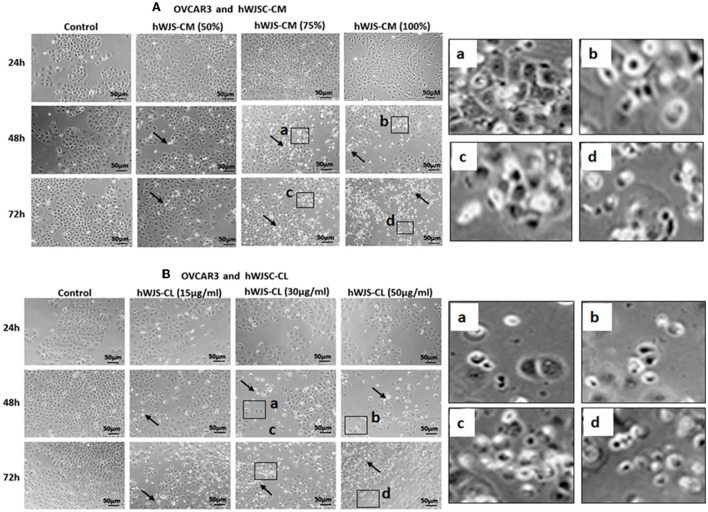
Representative phase contrast images of OVCAR3 cells treated with hWJSC-CM **(A)** and OVCAR3 cells treated with hWJSC-CL **(B)**. There were more cell death and decreases in OVCAR3 cells treated with increasing concentrations of hWJSC-CM and hWJSC-CL with time. Thin black arrows indicate dead translucent cells; The magnified images (a–d) represent the respective boxed area (a–d) within the main figure and shows decrease in the size of cells, membrane damages and condensed or fragmented nuclei. hWJSC-CM: human Wharton's jelly stem cells conditioned medium; hWJSC-CL: human Wharton's jelly stem cell lysate.

**Figure 5:**
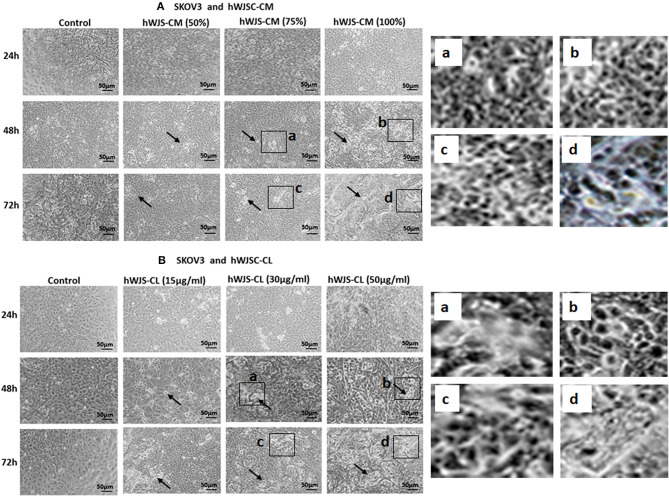
Representative phase contrast images of SKOV3 cells treated with hWJSC-CM **(A)** and SKOV3 cells treated with hWJSC-CL **(B)**. There were less cell death and decreases in SKOV3 cells treated with increasing concentrations of hWJSC-CM and hWJSC-CL with time. Thin black arrows indicate dead translucent cells; The magnified images (a–d) represent the respective boxed area (a–d) within the main figure and shows decrease in the size of cells, membrane damages and condensed or fragmented nuclei. hWJSC-CM, human Wharton's jelly stem cells conditioned medium; hWJSC-CL, human Wharton's jelly stem cell lysate.

OVCAR3 cells showed various morphological changes leading to cell death following treatment with hWJSC-CM (50, 75, 100 %) and hWJSC-CL (15, 30, 50 μg/ml) for 24, 48, and 72 h (Figures 4A,B). The morphological changes included cell shrinkage, membrane damage and cell death. Unlike OVCAR3 the SKOV3 cells showed few cellular changes leading to cell death following treatment with hWJSC-CM (50, 75, 100 %) and hWJSC-CL (15, 30, 50 μg/ml) (Figure 5A,B). In general, the cellular changes were both time and concentration dependent, and was more evident with higher concentrations of hWJSC-CM and hWJSC-CL.

### Cell Morphology of OVCAR3 and SKOV3 Cells With Paclitaxel and Doxorubicin

Both OVCAR3 (Supplementary Figures 1A,B) and SKOV3 (Supplementary Figures 2A,B) cells showed various morphological changes including cell shrinkage and membrane damage leading to cell death following treatment with pacliaxel (2.5, 5, 10, 20, 30 nM) and doxorubicin (3, 10, 30, 100, 300 nM) for 24, 48, and 72 h. The cellular changes were both time and concentration dependent (Supplementary Figures 1B, 2B).

### Cell Cycle Analysis of OVCAR3 and SKOV3 Cells With hWJSC-CM and hWJSC-CL

**Figure 6:**
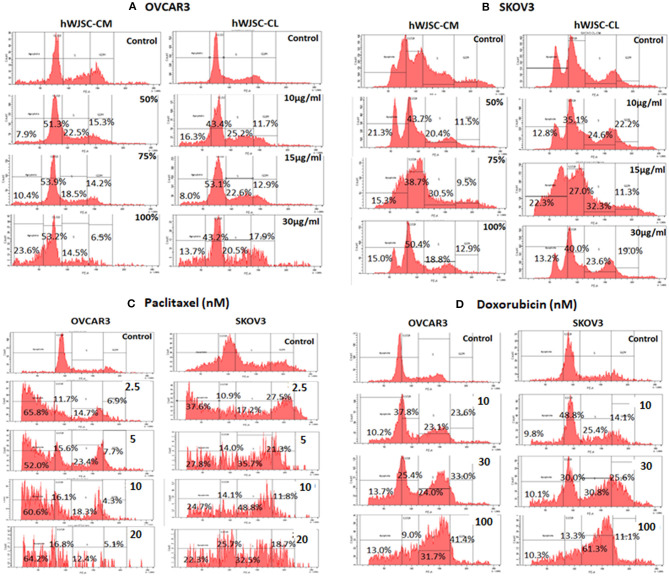
Cell cycle assay of **(A)** OVCAR3 cells treated with hWJSC-CM (left panel) and hWJSC-CL (right panel); **(B)** SKOV3 cells treated with hWJSC-CM (left panel) and hWJSC-CL (right panel); **(C)** OVCAR3 cells (left panel) and SKOV3 (right panel) cells treated with paclitaxel; **(D)** OVCAR3 cells (left panel) and SKOV3 (right panel) cells treated with doxorubicin. Treatment of both OVCAR3 and SKOV3 cells with hWJSC-CM (50, 75, 100%), hWJSC-CL (10, 15, 30 μg/ml), paclitaxel (2.5, 5, 10 nM), and doxorubicin (10, 30, 100 nM) for 48 h showed an increase in the sub-G1 and G2M phases of cell cylce indicative of apoptosis and metaphase arrest, respectively. The values are expressed as mean ± SEM of three different experiments.

Cell cycle assay of OVCAR3 and SKOV3 cells showed changes in the cell cycle profile following treatment with hWJSC-CM (50, 75, 100%) and hWJSC-CL (10, 15, 30 μg/ml) for 48 h, compared to the control. With OVCAR3 cells, there was an increase sub-G1 phase (by 7.9, 10.4, 26.3%) and G2M phase (by 15.3, 14.2, 6.5%) following treatment with hWJSC-CM (Figure 6A, Left panel). Treatment with hWJSC-CL demonstrated an increase in the sub-G1 population (by 16.3, 8.0, 13.7%) and the G2M phase (by 11.7, 12.9, 17.9%) compared to the control (Figure 6A, Right panel). With SKOV3 cells, there was an increase sub-G1 phase (by 21.3, 15.3, 15.0%) and G2M phase (by 11.5, 9.5, 12.9%) following treatment with hWJSC-CM (Figure 6B, Left panel. Treatment with hWJSC-CL demonstrated an increase in the sub-G1 population (by 12.8, 22.3, 13.2%) and the G2M phase (by 22.2, 11.3, 19.0%) compared to the control (Figure 6B, Right panel).

### Cell Cycle Analysis of OVCAR3 and SKOV3 Cells With Paclitaxel and Doxorubicin

Cell cycle assay of both OVCAR3 and SKOV3 cells at 48 h, showed changes in the cell cycle profile following treatment with paclitaxel (2.5, 5, 10 nM) and doxorubicin (10, 30, 100 nM) compared to the control. The OVCAR3 cells treated with paclitaxel showed an increase sub-G1 phase (by 65.8, 52.0, 60.6%) and G2M phase (by 6.9, 7.7, 4.3%) compared to the control (Figure 6C, Left panel). SKOV3 cells treated with paclitaxel also demonstrated an increase in the sub-G1 population (by 37.6, 22.8, 4.7%) and the G2M phase (by 27.5, 21.3, 11.8%) compared to the control (Figure 6C, Right panel). The OVCAR3 cells treated with doxorubicin showed an increase sub-G1 phase (by 10.2, 13.7, 13.0%) and G2M phase (by 23.6, 33.0, 41.4%) compared to the control (Figure 6D, Left panel). SKOV3 cells also demonstrated an increase in the sub-G1 population (by 9.8, 10.1, 10.3%) and the G2M phase (by 14.1, 25.6, 11.1%) compared to the control (Figure 6D, Right panel). Treatment of both OVCAR3 and SKOV3 cells with hWJSC extracts (hWJSC-CM, hWJSC-CL) as well as the standard anticancer agents (paclitaxel and doxorubicin) demonstrated increases in sub-G1 and the G2M phases of cell cycle compared to the control, indicating that the cancer cells underwent cell death *via* apoptosis and metaphase arrest.

### Caspase Activation Assay

**Figure 7:**
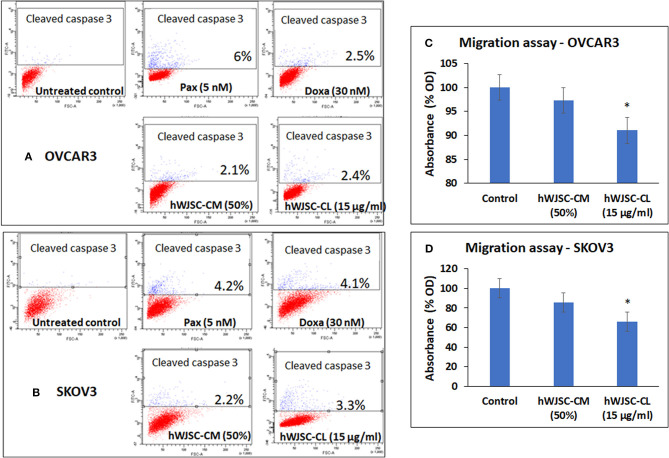
Representative scatter plots of Caspase 3 assay in **(A)** OVCAR3 and **(B)** SKOV3 cells follwing treatment with hWJSC-CM (50%),hWJSC-CL (15 μg/ml), paclitaxel (5 nM) and doxorubicin (30 nM) for 24 h. Mild expression of activated caspase 3 activity was observed in the treated group compared to the control following staining with FITC conjugated rabbit anti-caspase 3 antibody. Cell migration of **(C)** OVCAR3 and **(D)** SKOV3 following treatment with hWJSC-CM (50%) and hWJSC-CL (15 μg/ml) for 48 h. Decrease in cell migration was observed only with and hWJSC-CL (15 μg/ml) in both OVCAR3 and SKOV3 cells. The values are expressed as mean ± SEM of three different experiments. *indicates statistical significance (*p* < 0.05).

Both OVCAR3 and SKOV3 cells treated with hWJSC-CM (50%), hWJSC-CL (15 μg/ml), paclitaxel (5 nM) and doxorubicin (30 nM) showed mild positive expression of activated caspase 3 compared to the control (Figures 7A,B). The percentage of cells positve for activated caspase 3 with OVCAR3 cells were 6.0, 2.5, 2.1, and 2.4% following treatment with paclitaxel (5 nM), doxorubicin (30 nM), hWJSC-CM (50%), hWJSC-CL (15 μg/ml), respectively (Figure 7A). The percentage of cells positve for activated caspase 3 with SKOV3 cells were 4.2, 4.1, 2.2, and 3.3% compared to the untreated control (Figure 7B).

### Cell Migration Assay of OVCAR3 and SKOV3 Cells

The cell migration assay done on both OVCAR3 and SKOV3 cells following treatment with hWJSC-CM (50%) or hWJSC-CL (15 μg/ml) for 48 h showed that the hWJSC extracts (hWJSC-CM and hWJSC-CL) inhibited cancer cell migration compared to the control. The percentage decreases in migration observed with OVCAR3 and SKOV3 cells were 2.67, 8.97, and 17.32% and 34.20% for hWJSC-CM and hWJSC-CL, respectively (Figures 7C,D). However, only those decreases observed in OVCAR3 and SKOV3 cells with hWJSC-CL were statistically significant (*P* < 0.05).

### Tumor Sphere Assay of OVCAR3 and SKOV3 With hWJSC-CM and hWJSC-CL

**Figure 8:**
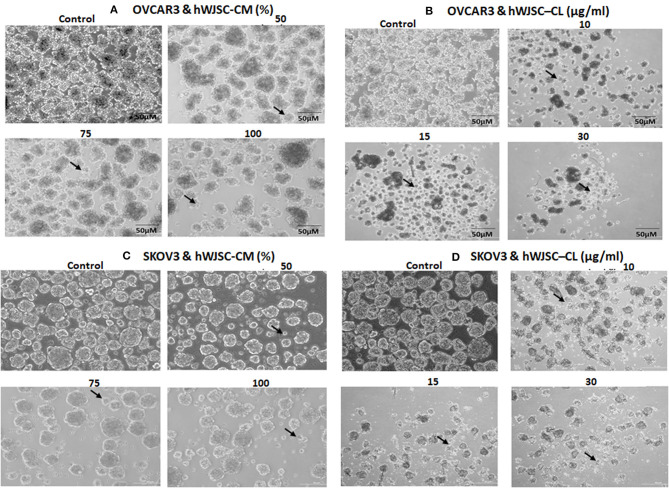
Representative phase contrast images of the tumor spheres (TS) of **(A)** OVCAR3 treated with hWJSC-CM; **(B)** TS of OVCAR3 treated with hWJSC-CL; **(C)** TS of SKOV3 treated with hWJSC-CM and **(D)** TS of SKOV3 treated with hWJSC-CL. Decrease in TS size, numbers and cell death/degeneration (indicated by thin black arrows) were more evident following treatment with hWJSC-CL (10, 15, and 30 μg/ml) than with hWJSC-CM (50, 75, and 100%) for 48 h.

The tumor spheres of both OVCAR3 and SKOV3 treated with hWJSC-CM (50, 75, 100 %) or hWJSC-CL (10, 15, 30 μg/ml) for 48 h showed changes in morphology, decrease in the number and size of TS compared to the control (Figures 8A–D). The morphological changes were more pronounced following treatment with hWJSC-CL, which showed more cell debris and cell death than hWJSC-CM in both OVCAR3 and SKOV3.

### Tumor Sphere Assay of OVCAR3 and SKOV3 With Paclitaxel and Doxorubicin

**Figure 9:**
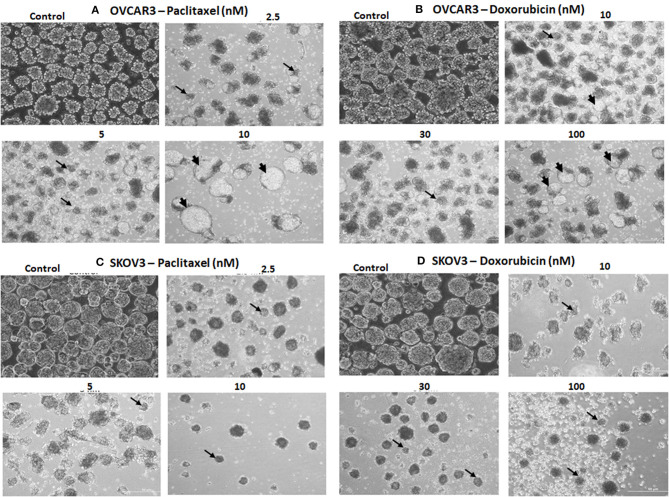
Representative phase contrast images of the tumor spheres (TS) of **(A)** OVCAR3 treated with paclitaxel; **(B)** TS of OVCAR3 treated with doxorubicin; **(C)** TS of SKOV3 treated with paclitaxel and **(D)** TS of SKOV3 treated with doxorubicin. Decrease in TS size, numbers and cell death/degeneration were more evident following treatment with paclitaxel (2.5, 5, 10 nM) than with doxorubicin (10, 30, 100 nM) for 48 h. Thin black arrows indicate the decrease in size of TS, while thick black arrow heads indicate the cystic degenerative changes noted with OVCAR3 tumor spheres.

The TS of both OVCAR3 and SKOV3 treated with paclitaxel (2.5, 5, 10 nM) and doxorubicin (10, 30, 100 nM) for 48 h also showed changes in morphology, decrease in the number and size of TS compared to the control (Figures 9A–D). More cystic degeneration was evident in OVCAR3 following treatment with both paclitaxel and doxorubicin. In contrast, there were more cell death and decrease in the sizes of TS in SKOV3 following treatment with both paclitaxel and doxorubicin.

## Cancer Stem Cell (CSC) Analysis of OVCAR3 and SKOV3 TUMOR Spheres (TS) Treated With hWJSC-CM and hWJSC-CL

**Figure 10:**
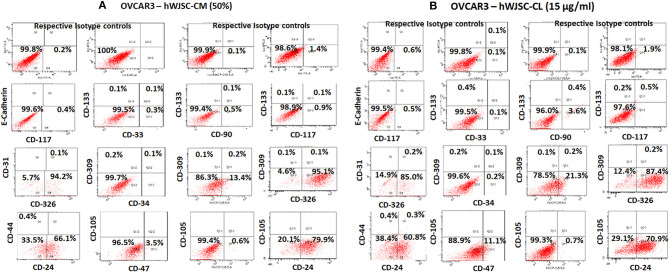
Representative histogram of the cancer stem cells (CSC) related CD surface markers in tumor spheres (TS) of **(A)** OVCAR3 treated with hWJSC-CM (50%) and **(B)** TS of OVCAR3 treated with hWJSC-CL (15 μg/ml using flrorescent activated cell sorting (FACS). Positive expression for some of the CSC markers were observed in the TS of OVCAR3 treated with hWJSC-CM and hWJSC-CM for 48 h.

The TS of OVCAR3 and SKOV3 treated with hWJSC extracts for 48 h demonstrated the presence of few CD markers which are reported to be CSC markers. TS of OVCAR3 treated with hWJSC-CM (50%) were minimally positive for CD117 (0.4–0.9%), CD133 (0.1%), CD33 (0.3%), CD309 (0.1–0.2%%), CD44 (0.4%), CD47 (3.5%), CD90 (0.5%), CD24 (66.1–79.9%), and CD326 (94.2–95.1%) (Figures 10A). TS of OVCAR3 treated with hWJSC-CL (15 μg/ml) were also minimally positive for CD117 (0.5%), CD133 (0.2–0.4%), CD309 (0.1–0.2%%), CD33 (0.1%), CD34 (0.2%), CD44 (0.4%), CD47 (11.1%), CD90 (3.6%), CD24 (60.8–70.9%), and CD326 (85.0–87.4%) (Figure 10B). TS of OVCAR3 treated with both hWJSC-CM and hWJSC-CL were negative for E-Cadherin, CD31 and CD105.

**Figure 11:**
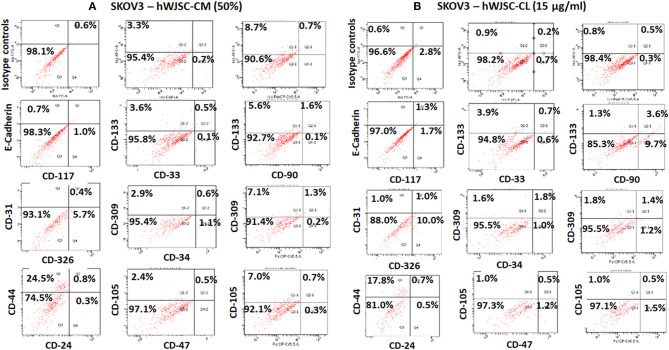
Representative histogram of the cancer stem cells (CSC) related CD surface markers in tumor spheres (TS) of **(A)** SKOV3 treated with hWJSC-CM (50%) and **(B)** TS of SKOV3 cells treated with hWJSC-CL (15 μg/ml using flrorescent activated cell sorting (FACS). Positive expression for some of the CSC markers were observed in the TS of SKOV3 treated with hWJSC-CM and hWJSC-CM for 48 h.

The TS of SKOV3 treated with hWJSC-CM (50%) were positive for E-Cadherin (0.7%), CD117 (1.0%), CD133 (3.6–5.6%), CD 33 (0.1%), CD31 (0.8%), CD309 (2.9–7.1%), CD326 (5.7%), CD34 (1.1%), CD44 (24.5%), CD90 (0.1%), CD24 (0.3%), and CD105 (2.4–7.0%). TS of SKOV3 treated with hWJSC-CM was negative for CD47 (Figure 11A). SKOV3 treated with hWJSC-CL (15 μg/ml) were positive for CD117 (1.7%), CD133 (1.3–3.9%), CD 33 (0.6%), CD31 (1.0%), CD309 (1.6–1.8%%), CD326 (10%), CD34 (1.0%), CD44 (17.8%), CD90 (9.7%), CD24 (0.5%), CD105 (1.0%), and CD47 (1.2%). TS of SKOV3 treated with hWJSC-CL was negative for E-Cadherin (Figure 11B).

### Cancer Stem Cell (CSC) Analysis of OVCAR3 and SKOV3 Treated With Paclitaxel

The TS of OVCAR3 treated with paclitaxel (10 nM) for 48 h were positive for CD117 (0.2–0.9%), CD133 (0.2%), CD33 (0.2%), CD309 (0.2%), CD44 (0.2%), CD47 (7.8%), CD90 (0.3%), CD24 (66.0–75.3%), CD105 (0.1%), and CD326 (95.1–96.0%). OVCAR3 treated with paclitaxel were negative for E-Cadherin and CD31 (Supplementary Figure 3A). SKOV3 treated with paclitaxel (10 nM) were positive for CD117 (1.4%), CD133 (2.4–5.7%), CD 33 (0.1%), CD31 (0.3%), CD309 (1.8–5.0%%), CD326 (8.9%), CD34 (0.2%), CD44 (28.5%), CD24 (0.2%), CD105 (1.6–5.3%), and CD47 (0.4%). SKOV3 treated with paclitaxel was negative for E-Cadherin and CD90 (Supplementary Figure 3B).

Screening of TS of OVCAR3 and SKOV3 for CSCs following treatment with hWJSC-CM (50%) and hWJSC-CL (15 μg/ml) and paclitaxel (10 nM) demonstrated minimal presence of some of the CD markers reported to be CSC markers associated with ovarian cancers. There were also differences in the expression pattern of CD markers between OVCAR3 and SKOV3.

### Quantitative Real-Time Gene Expression (qRT-PCR) Analysis

**Figure 12:**
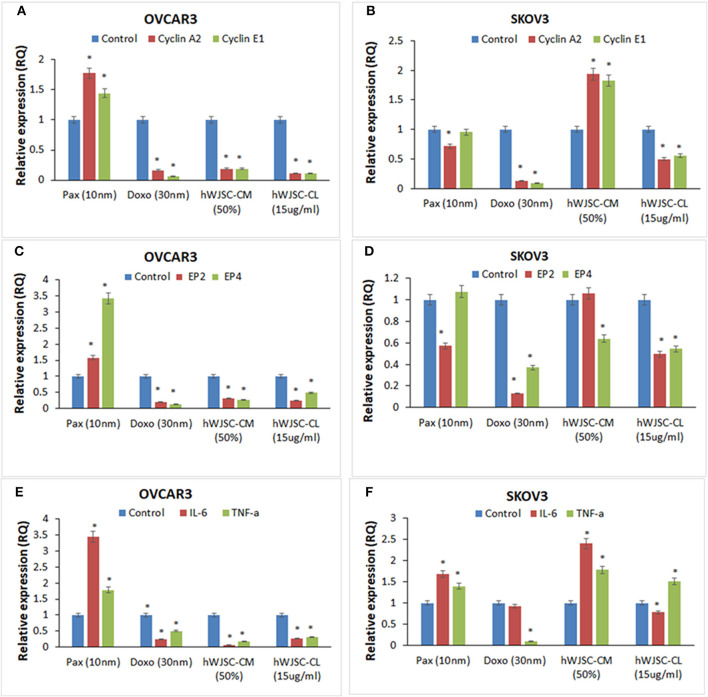
Gene expression analysis of cyclin A2 and cyclin E1 in OVCAR3 and SKOV3 cells **(A,B)**; EP2 and EP4 in OVCAR3 and SKOV3 cells **(C,D)**, and IL-6 and TNF-α in OVCAR3 and SKOV3 cells **(E,F)** using quantitative real-time PCR. Both OVCAR3 and SKOV3 treated with paclitaxel (10 nM), doxorubicin (30 nM), hWJSC-CM (50%) and hWJSC-CL (15 μg/ml) for 48 h showed decreased expression of cell cycle, prostaglandin receptor and inflammation related genes compared to control. Data analysis and relative quantitation were performed using the comparative Ct method (ΔΔCt). The differences in gene expression levels were analyzed using student's *t*-test. Asterisks (*) indicate statistical significance at *p* < 0.05.

The gene expression analysis done on both OVCAR3 and SKOV3 following treatment with hWJSC-CM (50%) or hWJSC-CL (15 μg/ml) for 48 h showed differential expression of the cell cycle regulatory genes (cyclin A2, Cyclin E1), prostaglandin receptors (EP2, EP4) and pro-inflammatory genes (IL-6, TNF-a) (Figures 12A–F).

### Cyclin A2 and Cyclin E1 Genes Expression in OVCAR3 and SKOV3 Cells

OVCAR3 cells demonstrated decreases in cyclin A2 and cyclin E1 expression with doxorubicin (30 nM) (by 83.45, 93.61%); hWJC-CM (by 81.06, 80.91%); and hWJSC-CL (by 89.38, 89.16%), respectively. In contrast, treatment with paclitaxel (10 nM) demonstrated increase in cyclin A2 and cyclin E1 expression by 77.33 and 44.31% (Figure 12A). Treatment of SKOV3 cells with hWJC-CM demonstrated an increase in cyclin A2 and cyclin E1 expression (by 93.53 and 82.37%), while there were decreases with paclitaxel (by 28.27, 4.64%), doxorubicin (by 87.17, 90.45%) and hWJSC-CL (by 49.44, 44.24%), respectively (Figure 12B).

### EP2 and EP4 Genes Expression in OVCAR3 and SKOV3 Cells

Treatment of OVCAR3 cells with paclitaxel demonstrated increases in EP2 and EP4 genes expression by 58.00 and 242.72%. In contrast, decreases in EP2 and EP4 expression were observed with doxorubicin (by 78.98%,85.94%); hWJC-CM (by 65.89, 73.41%); and hWJSC-CL (by 75.31, 51.24%), respectively (Figure 12C). Differential expression of EP2 and EP4 were observed following treatment of SKOV3 cells with paclitaxel (decrease in EP2 by 42.70% and an increase in EP4 by 7.63%) and hWJSC-CM (increase in EP2 by 6.16% and a decrease in EP4 by 36.03%). However, treatment with doxorubicin and hWJSC-CL for 48 h deomonstrated decreases in EP2 and EP4. The percentage decreases were 87.00%, 62.97% with doxorubicin and 50.49%, 45.42% with hWJSC-CL, respectively (Figure 12D).

### IL-6 and TNF- αGenes Expression in OVCAR3 and SKOV3 Cells

Treatment of OVCAR3 cells with paclitaxel (10 nM) for 48 h demonstrated increases in IL-6 and TNF-α genes expression by 243.07 and 77.78%. In contrast, decreases of both IL-6 and TNF-α expression were observed with doxorubicin (30 nM) by 75.47, 49.35%; hWJC-CM (50%) by 95.35, 82.86%; and hWJSC-CL (15 μg/ml) by 72.53, 68.10%, respectively (Figure 12E). Treatment of SKOV3 with paclitaxel and hWJC-CM for 48 h demonstrated increases in IL-6 and TNF-α genes expression by 68.57, 39.62 and 141.22, 78.34%, respectively. Treatment of SKOV3 cells with doxorubicin demonstrated decreases in IL-6 and TNF-α genes expression by 6.94 and 89.77%, respectively. However, treatment of SKOV3 cells with hWJSC-CL demonstrated differential expression (a decrease in IL-6 by 21.64% and an increase in TNF-α by 51.03%) (Figure 12F).

## Discussion

Mesenchymal stem cells (MSCs) including the hWJSCs derived from within the umbilical cord have been used as anticancer agents either naïve or engineered against various human cancers (20–22). The hWJSCs have many advantages compared to other currently available MSCs such as painless harvest as they are derived from umbilical cord which are usually discarded at birth, relatively young when derived, highly proliferative, have long telomeres and widely multipotent (14). In the present study, freshly prepared hWJSC-CM and hWJSC-CL inhibited growth and metabolic activity of both OVCAR3 and SKOV3 cells *in vitro*. The inhibition in growth and metabolic activity could partly be explained by the cellular damage induced by hWJSC extracts on OVCAR3 (Figures 4A,B) and SKOV3 (Figures 5A,B) cells eventually leading to cell death. The morphological changes such as cell shrinkage, membrane blebbings leading to cell death were indicative of apoptosis. Furthermore, the cell cycle analyses revealed increased population of cells in the sub-G1 phase which indicate that most of the ovarian cancer cells underwent cell death *via* apoptosis (Figures 6A,B). In addition, the cell cycle assay also showed that both OVCAR3 and SKOV3 cells underwent metaphase arrest (Figures 6A,B). Also cleaved caspase 3 levels were mildly increased following treatment of OVCAR3 and SKOV3 cells with hWJSC extracts (Figure 7A,B). Therefore, the observed inhibition of growth and metabolic activity of the ovarian cancer cells in the present study is in part due to cell cycle inhibition and apoptosis. Earlier studies using MSCs against cancer cell inhibition have reported similar morphological changes leading to cell death (11, 12, 16, 23).

Abnormalities in the cell cycle regulatory pathways involving oncogenes and tumor suppressor genes is associated with cancer initiation and growth. Control of the various phases of cell cycle appear to limit progression of various cancers. Many small molecules including phytochemicals have demonstrated effective cell cycle inhibition. Cucurbitacin B induced G2M arrest and apoptosis in paclitaxel resistant ovarian cancer cells by increasing expression of cell cycle regulators p21 and p53 (24). Also cell cycle inhibition in human ovarian cancer cell lines SKOV3 and OVCA-420 was reported with transfection of cell cycle regulators p16, p21 and p53 using adenoviral vectors (25). Interestingly, in line with our findings G2M arrest and apoptosis in ovarian cancer cells with hWJSCs, the MSCs of the endometrium were reported to inhibit ovarian tumor growth by induction of cell cycle arrest and apoptosis (26). Also the human umbilical cord derived MSCs have been earlier reported to induce apoptosis of glioma and xenografted cells via activation of caspase 3 and caspase 9 (27).

We also identified that the hWJSC-CM and hWJSC-CL decreased OVCAR3 and SKOV3 cell migration (Figures 7C,D). This indicates that the hWJSC extracts had the potential to inhibit ovarian cancer cell motility which can be helpful to prevent metastasis. (28) reported that the conditioned medium derived from MSCs inhibited migration of lung cancer cells (A549) by decreasing the phosphorylation of human epidermal growth factor 3 (28). It is possible that hWJSC-CM and hWJSC-CL inhibited ovarian cancer cell migration by similar mechanism.

Treatment with hWJSC-CM and hWJSC-CL led to reduction in TS size compared to untreated controls probably by induction of necrosis/apoptosis. However, TS continued to survive and were not fully ablated and this can either be due to (i) insufficient concentration of hWJSC-CM and hWJSC-CL, (ii) decreased contact period or (iii) due the presence of few resistant stem cells within these tumor spheroids. Screening TS for a battery of CD markers including CSC markers following treatment with hWJSC-CM and hWJSC-CL demonstrated minimal expression of some of the CSC markers reported in ovarian cancer (Figures 10A–D). Treatment with hWJSC extracts reduced TS size/numbers and thereby the expression levels of CSC markers indicating that hWJSC extracts may be helpful in inhibition or prevention of ovarian cancer relapse/recurrence. CSCs are known to impart chemoresistance and resist apoptosis, and their pattern of expression vary with different cancers. CD44, CD24, CD133, CD117, and EPCAM have been reported to be associated with ovarian cancer (29, 30). These markers are neither universal for all cancers and also they may be expressed in early progenitor cells. CD44 and CD133 are penta membrane glycoprotein and are usually associated with cell membrane structure and function. CD44, being a receptor for hyaluronate, selectin, fibronectin and osteopontin, mediates cell to cell adhesion and ECM interactions (31). CD133 (Prominin-1) is a neural stem cell marker and its expression is not restricted to CSCs alone. CD117 (c-kit), CD44, CD24, and CD133 are usually expressed in solid tumors including ovarian cancer (31). CD90 is reported to inhibit ovarian cancer formation by interaction with β3-integrin and it also reduces the expression of CD133 and CD24 (32). E-cadherin is expressed in malignant ovarian tissues but not in normal ovarian surface epithelium and is associated with cancer cell proliferation (33). CD31 is a pan endothelial marker and is associated with microvessel density, and increased expression levels is associated with poor prognosis (34). The significance of other CD markers identified in OVCAR3 and SKOV3 tumor spheres in relation to CSCs are unknown at present.

Gene expression analysis showed that inflammation related genes (IL-6 and TNF-α), the cell cycle regualtory genes (cyclin A2 and cyclin E1) as well as prostaglandin receptor genes (E2, E4) were downregulated with hWJSC extracts (Figures 12A–F). Cyclin A2 controls “S” phase and “G2/M” phase of cell cycle regulation in association with cyclin dependent kinases (cdk1 and cdk2) (35). Cyclin E1 overexpression is associated with increased “life time ovarian cycles” and ovarian cancer development is implicated with “incessant ovulation hypothesis”. Cyclin E1, is found to be overexpressed in various cancers including ovarian cancer and hence considered as a biomaker (36). Ovarian cancers share “estrogen regulated pathways” with “hormone related pathways” involved in other cancers. E2 is the active form of natural oestrogens and together with progesterone is involved in normal functioning of the uterus and associated glandular tissues. In an *in vivo* model of ovarian carcinoma with ER+ tumors, E2 induced progesterorne receptors, increased the size of tumors and promoted lymph node metastasis and E2 regulated genes expression (37). Prostaglandin receptors E2 and E4 play an important role in angiogenesis and in concert with vascular endothelial growth factor (VEGF) is involved in tumor progression and invasiveness (19). Cancer profiling array identified higher expression of TNF- α malignant ovarian tissue compared to normal ovarian tissue and also ovarian cancer cells compared to normal ovarian cells (38). Increased expression of CD9 in SKOV3 cells was associated with increased TNF-α gene expression and nuclear factor kappa B (NFk-B) signaling (39). IL-6 is a growth promoting and apoptotic factor. Interleukin 6 (IL-6) and its soluble receptor sIL-6R is expressed by various human carcinoma cell lines *in vitro*. IL-6 activates the signal transduction pathways and is associated with migration of ovarian and mesentry microvasuclar endothelial cells (40).

In conclusion, the results obtained in our studies indicate that the stem cell soluble factors either present within or largely secreted by hWJSCs may be useful in ovarian cancer inhibition. Furthermore, as there were no direct contact of the stem cells (hWJSCs) with OVCAR3 or SKOV3 cells, the inhibitory effects can be attributed solely to the presence of soluble factors in hWJSC extracts. In depth proteomics studies will help to identify the putative molecule(s) in hWJSC extracts which can help advance stem cell based cancer therapeutics.

## Author Contributions

GK was involved in conceptualization, intellectual contribution, statistical evaluation and manuscript writing. KS and NA are the clinicians and were involved in providing clinical materials/information and intellectual support. RK, FA, MJ were involved in assisting the experimental work, data analysis, manuscript editing and intellectual help. PP, MR, MA-E and MA-Q were involved in co-ordination of the work, and also reviewed and edited the manuscript.

### Conflict of Interest Statement

The authors declare that the research was conducted in the absence of any commercial or financial relationships that could be construed as a potential conflict of interest.

## Supplementary Material

Supplementary Figure 1 Representative phase contrast images of OVCAR3 cells treated with paclitaxel **(A)** and OVCAR3 cells treated with doxorubicin **(B)**. There were more cell death and decreases in OVCAR3 cells with increasing concentrations and time of the standard anti-cancer agents (paclitaxel and doxorubicin). Thin black arrows indicate dead translucent cells.

Supplementary Figure 2 Representative phase contrast images of SKOV3 cells treated with paclitaxel **(A)** and SKOV3 cells treated with doxorubicin **(B)**. There were less cell death and decreases in SKOV3 cells with increasing concentrations and time of the standard anti-cancer agents (paclitaxel and doxorubicin). Thin black arrows indicate dead translucent cells.

Supplementary Figure 3 Representative histogram of the cancer stem cells (CSC) related CD surface markers in tumor spheres (TS) of **(A)** OVCAR3 cells treated with paclitaxel (10 nM) and **(B)** TS of SKOV3 cells treated with paclitaxel (10 nM) using flrorescent activated cell sorting (FACS). Positive expression for some of the CSC markers were observed in both the TS of OVCAR3 and SKOV3 treated with the standard anticancer agent paclitaxel (10 nM) for 48 h.
