# Corrigendum: Human Wharton’s Jelly stem cell (hWJSC) extracts inhibit ovarian cancer cell lines OVCAR3 and SKOV3 *in vitro* by inducing cell cycle arrest and apoptosis

**DOI:** 10.3389/fonc.2023.1171430

**Published:** 2023-03-20

**Authors:** Gauthaman Kalamegam, Khalid Hussein Wali Sait, Farid Ahmed, Roaa Kadam, Peter Natesan Pushparaj, Nisreen Anfinan, Mahmood Rasool, Mohammad Sarwar Jamal, Muhammed Abu-Elmagd, Mohammed Al-Qahtani

**Affiliations:** ^1^ Center of Excellence in Genomic Medicine Research, King Abdulaziz University, Jeddah, Saudi Arabia; ^2^ Faculty of Medicine, Asian Institute of Medicine, Science and Technology (AIMST) University, Bedong, Malaysia; ^3^ Department of Obstetrics and Gynaecology, Faculty of Medicine, King Abdulaziz University, Jeddah, Saudi Arabia; ^4^ King Fahad Medical Research Centre (KFMRC), King Abdulaziz University, Jeddah, Saudi Arabia

**Keywords:** stem cells, cancer stem cells, cell cycle, cell migration, tumor spheres, gene expression, OVCAR3, SKOV3

**Error in Figure**

In the published article, there was an error in **Figure 4A** as published. In **Figure 4A**, the image representing hWJS-CM (100%) at 24h was identified to be the same as the hWJS-CM (50%) at 48h. The corrected **Figure 4A** and its caption appear below.

**Figure 4:**
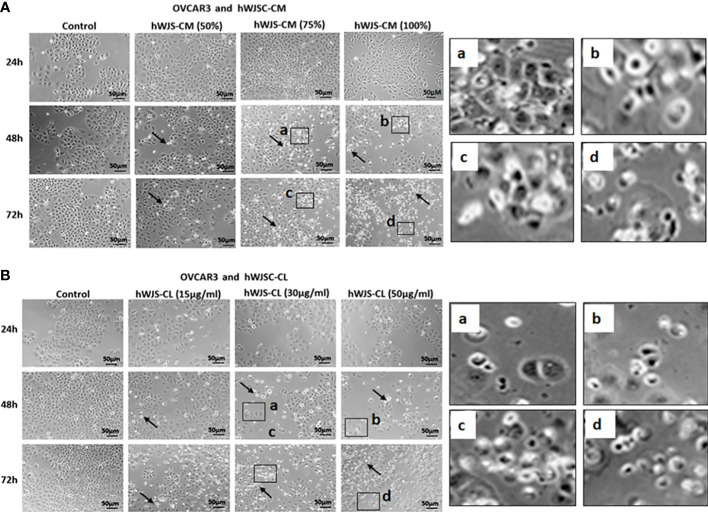
Representative phase contrast images of OVCAR3 cells treated with hWJSC-CM **(A)** and OVCAR3 cells treated with hWJSC-CL **(B)**. There were more cell death and decreases in OVCAR3 cells treated with increasing concentrations of hWJSC-CM and hWJSC-CL with time. Thin black arrows indicate dead translucent cells; The magnified images (a–d) represent the respective boxed area (a-d) within the main figure and shows decrease in the size of cells, membrane damages and condensed or fragmented nuclei. hWJSC-CM: human Wharton’s jelly stem cells conditioned medium; hWJSC-CL, human Wharton’s jelly stem cell lysate.

The authors apologize for this error and state that this does not change the scientific conclusions of the article in any way. The original article has been updated.

