# Supplementary figures and images for: Human Wharton's Jelly Stem Cell (hWJSC) Extracts Inhibit Ovarian Cancer Cell Lines OVCAR3 and SKOV3 in vitro by Inducing Cell Cycle Arrest and Apoptosis
Source: Front Oncol. 2018 Dec 7;8:592. doi: 10.3389/fonc.2018.00592 (PMC6293270; doi:10.3389/fonc.2018.00592)

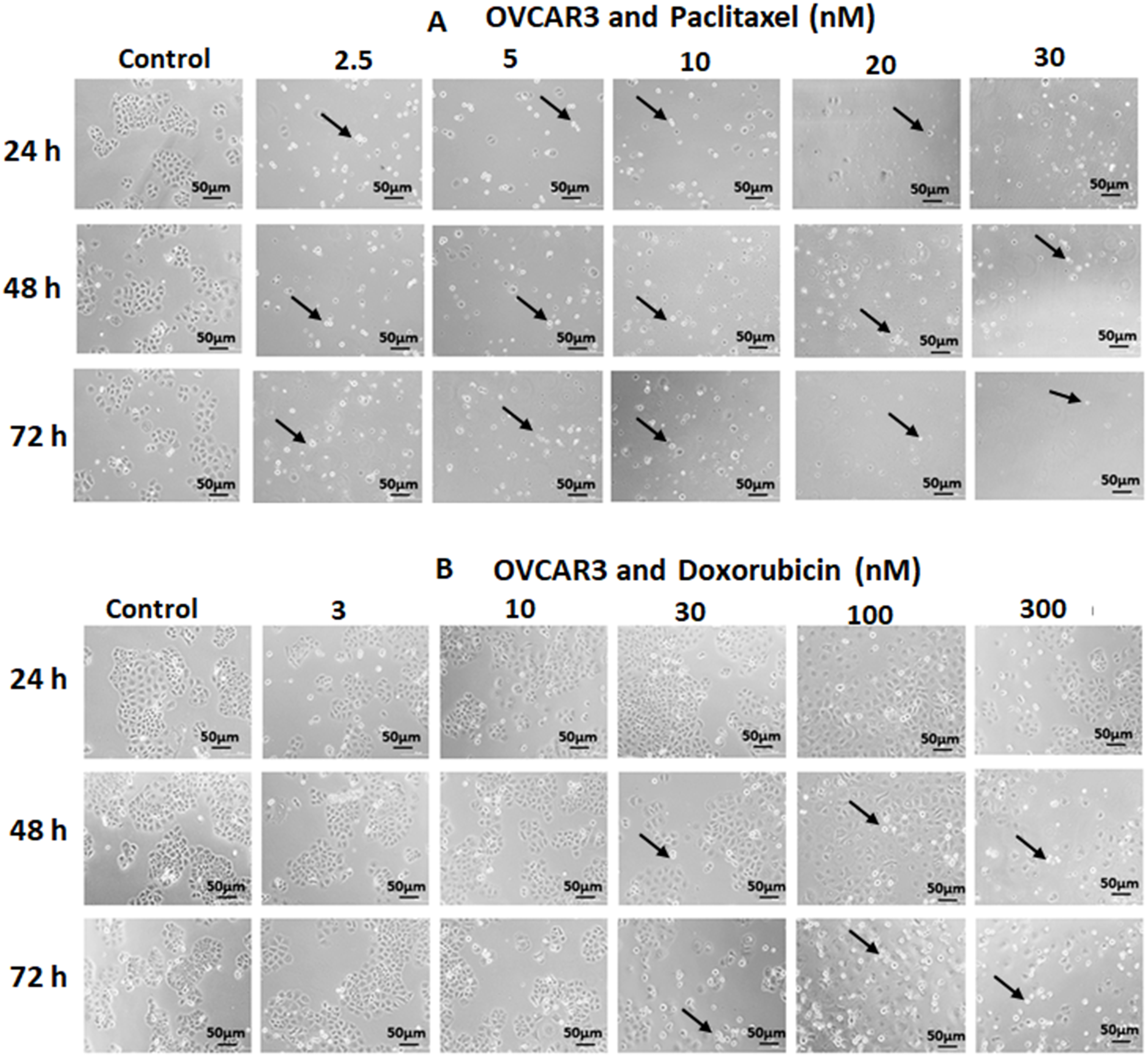

Supplement: Supplementary Figure 1 — Representative phase contrast images of OVCAR3 cells treated with paclitaxel (A) and OVCAR3 cells treated with doxorubicin (B). There were more cell death and decreases in OVCAR3 cells with increasing concentrations and time of the standard anti-cancer agents (paclitaxel and doxorubicin). Thin black arrows indicate dead translucent cells. [file Image_1.TIF]

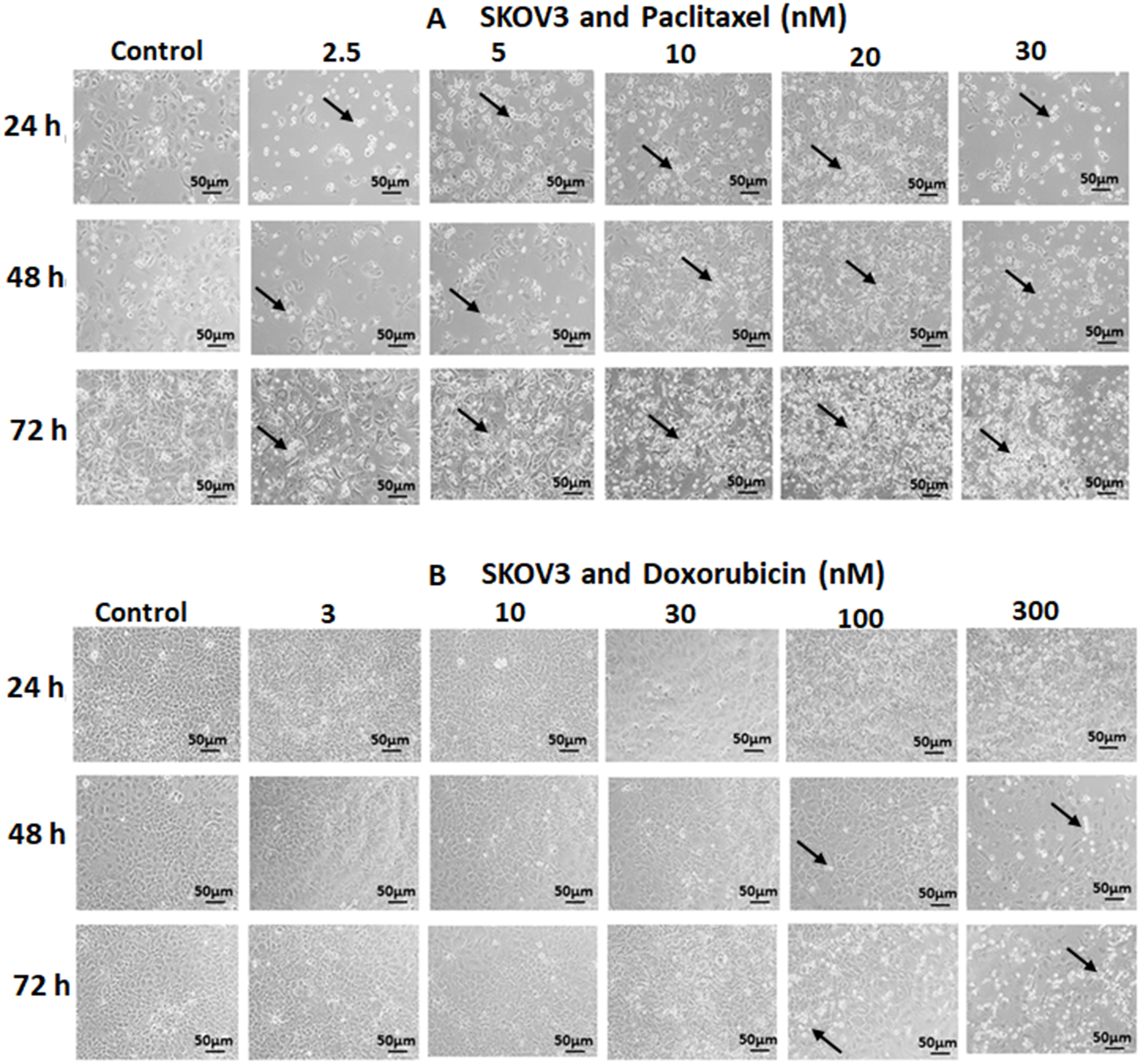

Supplement: Supplementary Figure 2 — Representative phase contrast images of SKOV3 cells treated with paclitaxel (A) and SKOV3 cells treated with doxorubicin (B). There were less cell death and decreases in SKOV3 cells with increasing concentrations and time of the standard anti-cancer agents (paclitaxel and doxorubicin). Thin black arrows indicate dead translucent cells. [file Image_2.TIF]

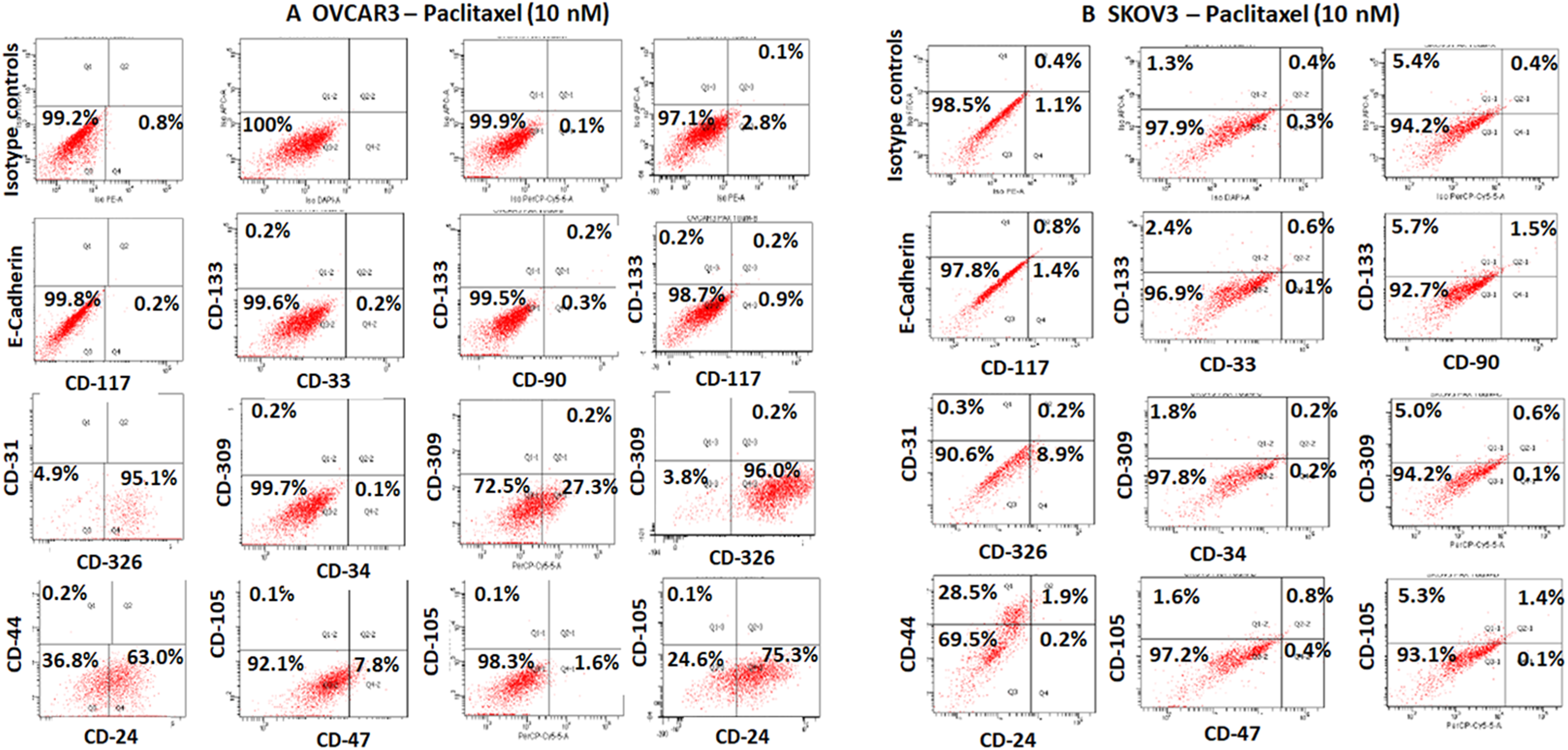

Supplement: Supplementary Figure 3 — Representative histogram of the cancer stem cells (CSC) related CD surface markers in tumor spheres (TS) of (A) OVCAR3 cells treated with paclitaxel (10 nM) and (B) TS of SKOV3 cells treated with paclitaxel (10 nM) using flrorescent activated cell sorting (FACS). Positive expression for some of the CSC markers were observed in both the TS of OVCAR3 and SKOV3 treated with the standard anticancer agent paclitaxel (10 nM) for 48 h. [file Image_3.TIF]
